# Blood-based biomarkers in centenarians and non-centenarians: a matched, population-based retrospective cohort study using primary care records in Catalonia, Spain

**DOI:** 10.1007/s10522-025-10258-3

**Published:** 2025-05-26

**Authors:** Manuel A. Moreno, Josep Vidal-Alaball, Marc Saez, Maria A. Barceló

**Affiliations:** 1 Research Group On Statistics, Econometrics and Health (GRECS), University of Girona, Carrer de La Universitat de Girona 10, Campus de Montilivi, 17003 Girona, Spain; 2 Institut Català de La Salut. Gerència d’Atenció Primària I a La Comunitat de La Catalunya Central, Unitat de Recerca I Innovació, 08242 Manresa, Spain; 3 Centro de Investigación Biomédica en Red de Epidemiología y Salud Pública, Instituto de Salud Carlos III, Madrid, Spain; 4 Intelligence for Primary Care Research Group, Foundation University Institute for Primary Health Care Research Jordi Gol I Gurina, 08242 Manresa, Spain; 5 Faculty of Medicine, University of Vic – Central University of Catalonia, Vic,, Barcelona Spain

**Keywords:** Centenarians, COVID-19, Blood-based biomarkers, Contextual level, Spatial dependence

## Abstract

The global increase in life expectancy has sparked growing interest in the factors that contribute to exceptional longevity. Between 1990 and 2015, the number of centenarians worldwide more than quadrupled. This study aimed to analyse the relationship between blood-based biomarkers and the likelihood of reaching 100 years of age in Catalonia (2015–2022), and to examine how biomarker variations during COVID-19 affected longevity. Using a retrospective cohort study based on primary care electronic health records from Catalonia, we compared centenarians with individuals aged 92 or older who died before reaching 100 years of age. We analysed anaemia, cholesterol, glycemia, kidney function, and liver function biomarkers. We employed multiple strategies to control for confounding including matching without replacement, adjusting for both observed confounders at both the individual and contextual level, and unobserved confounders, in particular spatial dependence. Our findings reveal that centenarians exhibit higher rates of chronic conditions, greater socioeconomic disadvantage, and increased neighbourhood inequality in urban areas. Biologically, longevity was linked to intermediate levels of ferritin and cholesterol, alongside lower glucose, creatinine, and uric acid levels. Glycaemic balance, indicated by HbA1c and fasting glucose, emerged as a key factor in survival to extreme old age. Additionally, biomarker improvements during the pandemic correlated with an increased likelihood of reaching centenarian age. These results emphasize the complex interplay between biological, behavioural, and contextual factors in determining longevity. While biomarkers provide valuable insights, they are insufficient indicators of healthy ageing. Future research should integrate multiple dimensions, among them, environmental, and social determinants for uncovering the mechanisms of longevity.

**Supplementary Information:**

The online version contains supplementary material available at 10.1007/s10522-025-10258-3.

## Introduction

The global increase in life expectancy has sparked growing interest in understanding the factors that contribute to reaching very advanced ages. According to United Nations estimates, in 2015 there were nearly half a million people who had reached the age of 100 years or more in the world, more than four times the number of centenarians in 1990 (United Nations 2024). The projections predict that there will be 3.7 million centenarians in the world by 2050 (United Nations 2024). This trend is particularly evident in industrialized countries, where advances in healthcare, social policies, and improvements in quality of life have led to a growing proportion of the population living beyond this milestone. Given the rapid ageing of the population and the growing need to understand how to live longer in good health, it is no surprise that research has increasingly focused on centenarians and supercentenarians (those who have lived at least 110 years), exploring the biological, genetic, social and environmental factors that may have contributed to their longevity.

According to Islam et al*.*, centenarians are a group of survivors who have escaped or considerably delayed the onset of diseases that normally claim the lives of their peers at much earlier ages, especially those with high morbidity and mortality, such as cardiovascular diseases, neurodegeneration and cancer (Islam et al. 2024). In this regard, most studies focus on specific health outcomes such as cognitive function, disabilities, comorbidities and hospitalizations (Engberg et al. 2009; Ismail et al. 2016; Vetrano et al. 2021). Few, however, evaluate the role that blood-based biomarkers play in reaching centenarian status (Barzilai et al. 2003; Milman et al. 2014; Ding et al. 2021; Murata et al. 2024) and even fewer study the role of changes in these biomarkers (Ding et al. 2021; Murata et al. 2024). Blood-based biomarkers can provide information about adverse health outcomes before they even occur (Murata et al. 2024). Furthermore, comparing the levels of biomarkers in centenarians with those of individuals who did not reach 100 years of age, could offer valuable insights into the factors that contribute to exceptional longevity and the extent of their impact. Murata et al*.* suggest that extreme longevity may be associated with certain metabolic and biological characteristics that protect against chronic diseases (Murata et al. 2024).

On the other hand, the potential impact of the COVID-19 pandemic on the likelihood of becoming a centenarian has not yet been studied. In the general population, Moreno-Vásquez et al*.* found that the pandemic led to significant variations in the levels of some blood biomarkers, especially in chronically ill and vulnerable populations (Moreno-Vásquez et al. 2024). In this study, we hypothesize that these variations also occurred in individuals of advanced ages, and that these could have had some influence on the probability of being a centenarian.

We used a large population-based retrospective cohort for Catalonia. Catalonia, an autonomous community in northeastern Spain, presents an intriguing case for such analysis due to its sociocultural, environmental, and demographic characteristics Recent data shows that Spain ranks among the highest in life expectancy globally, and Catalonia has seen a notable rise in its centenarian population over the past few decades (Foreman et al. 2018). Catalonia’s centenarian population has increased 4.6 times from the start of the millennium to 2021. Between 2011 and 2021, both the number of centenarians and supercentenarians almost doubled – rising from 1359 (1.81 per 10,000 inhabitants) to 2280 (2.94 per 10,000 inhabitants), and from 65 to 130, respectively) (IDESCAT. Institut d’Estadística de Catalunya 2021). The Catalonian population exhibits distinctive traits, including a Mediterranean diet, high levels of social engagement, and a well-established healthcare system. However, there are differences in the distribution of centenarians across provinces, with some rural areas in Lleida (one of the four provinces into which Catalonia is divided) having higher rates of longevity compared to urban settings in other provinces (INE 2025a). Additionally, the role social factors play in longevity cannot be underestimated. Social isolation, which has been identified as a risk factor for premature mortality worldwide (Naito et al. 2023), appears to be less prevalent in Catalonia’s tightly knit communities, particularly in rural settings. Catalonia has a strong sense of community and family structure, both of which are known to contribute to longer and healthier lives. These disparities invite a closer examination of the contributing factors. While numerous studies have examined global factors contributing to longevity, there remains a gap in region-specific studies that focus on how local factors play a role in extreme ageing.

Our study had two objectives. First, to contribute to the existing body of knowledge by conducting a comprehensive analysis of the association between blood-based biomarkers and the likelihood of reaching centenarian age in Catalonia during the period 2015–2022. Second, to assess how variations in these biomarkers during the COVID-19 pandemic influenced the likelihood of reaching centenarian age.

There are several differences between our study and other similar studies, such as Arai et al. (2015), Ding et al. (2021), and Murata et al. (2024), and in Milman et al. (2014) and Barzilai et al. (2003). First, we followed different strategies to control for confounding. Thus, we matched centenarians with those individuals who, despite having the potential to reach 100 years of age in the follow-up period (2015–2022), died before achieving that milestone. Furthermore, unlike Barzilai et al. (2003), who also matched by age, we used numerous other variables for matching. In addition, we adjusted for many more confounders, both at the individual level and, unlike the abovementioned studies, at the contextual level. Furthermore, we are the only ones to adjust for observed confounders and unobserved ones. Notably, we controlled for spatial dependence, accounting for variables specific to the health area where an individual lived that may influence the probability of becoming a centenarian. Finally, no other studies have analysed the effect of biomarker variations during the COVID-19 pandemic on the probability of becoming a centenarian.

## Methods

We reported our study per the REporting of studies Conducted using Observational Routinely collected Data (RECORD) checklist (Benchimol et al. 2015), which is an extension of the STrengthening the Reporting of OBservational studies in Epidemiology (STROBE) guidelines (Von Elm et al. 2014) (Table S1 in the Supplementary Material).

### Study area and period

We conducted a retrospective observational population-based cohort study using primary care electronic health records from the Information System for Research in Primary Care (SIDIAP from its Catalan acronym *‘Sistema d’Informació per al Desenvolupament de la Investigació en Atenció Primària’*). The study covered the period from 1 January 2015 to 31 December 2022. SIDIAP is the largest primary care database in Catalonia, encompassing extensive information from 288 primary care centres managed by the Catalan Institute of Health (ICS from its Catalan acronym, *‘Institut Català de la Salut*’). By the end of 2022, the database included records for 6,473,566 individuals—representing 80.7% of the population of Catalonia (Moreno-Vásquez et al*.,* 2024; IDESCAT, 2021) – capturing data on demographics, visits, diagnoses, laboratory tests, and drug prescriptions (Recalde et al. 2022; Moreno-Vásquez et al*.,* 2024).

The selection criteria for this study were as follows: i) individuals born before 1 January 1923 and alive on 1 January 2015 were included, ensuring all participants had the potential to reach 100 years of age during the study period (2015–2022); ii) individuals must have resided in Catalonia at some point during the study period; iii) individuals were required to have undergone at least one blood test, with all the corresponding results recorded in the SIDIAP database. Individuals who complied with the selection criteria and died before their 100th birthday were classified as controls, and observed centenarians were classified as cases. The study period was divided into two phases for modelling: a pre-pandemic phase (before 1 April 2020) and a pandemic phase (1 April 2020 to 31 December 2022). The rationale for this division is discussed in subsequent sections.

### Variables

#### Outcome variable

The outcome variable was defined as an indicator for individuals who were either 100 years of age or older on January 1, 2015, or who turned 100 years old in the period 2015–2022 (cases from now on). Those who died before reaching 100 were classified as controls.

#### Explanatory variables

As explanatory variables we included: anaemia blood-based biomarkers (iron, haemoglobin and ferritin); cholesterol biomarkers (total cholesterol, high-density lipoprotein cholesterol -HDL-C- and low-density lipoprotein cholesterol -LDL-C-); glycemia biomarkers (fasting blood glucose and glycosylated haemoglobin -HbA1c-); kidney function biomarkers (glomerular filtration rate -CKD-EPI-, urea and creatinine); and a liver functioning biomarker (alkaline phosphatase -ALP-). All biomarkers were measured under fasting conditions.

To meet our first objective—assessing the influence of blood-based biomarkers on the likelihood of becoming a centenarian—biomarkers were categorized into quintiles. We used the biomarker values ​​corresponding to the most recent blood test taken before April 2020 (the declaration of the COVID-19 pandemic) to avoid any distortion that could have been caused by the pandemic.

To address our second objective—examining the association between biomarker variation during the pandemic and the probability of becoming a centenarian—we first calculated the change in biomarker values ​​by comparing pre-pandemic and pandemic-period measurements. We then derived a binary indicator of improvement based on the standardized change for each biomarker. To identify meaningful enhancement, we tested several cut-off points at different percentiles (25th/75th, 10th/90th, and 5th/95th). We selected the cut-off points after careful examination to preserve a substantial sample size and to ensure that the credibility intervals following model fitting were narrow enough to allow meaningful interpretation of the results. For example, the cut-off points at the 5th/95th and at the 10th/90th percentiles resulted in a small number of subjects showing improvement in biomarkers (see Table S7 in the supplementary material). Furthermore, the average width of the credibility intervals of the cut-off points at the 5th/95th percentiles was equal to 12.5; at the 10th/90th percentiles to 7.88; and at the 25th/75th percentiles equal to 5.14. Finally, we selected the cut-off points at the 25th/75th percentiles. Improvement was defined as a standardized change value below the 25th percentile of the distribution (i.e. standardized change < –0.674), indicating a favourable shift for glycaemic, cholesterol, kidney and liver function biomarkers. However, for HDL cholesterol, CKD-EPI, and anaemia-related biomarkers, where higher values ​​are generally considered beneficial, improvement was defined as a standardized change above the 75th percentile (i.e. standardized change > 0.674). Pre-pandemic values ​​correspond to the blood test result closest to the official start of the epidemic declaration, while pandemic-period values ​​were those recorded closest to December 31, 2022.

#### Covariates

Centenarians are known to constitute a very heterogeneous population due to a variety of factors, including lifestyle habits, environmental influences, genetics and dietary choices, all of which have shaped their biological and clinical profile differently throughout their lives (Islam et al. 2024; Vacante et al. 2012; Grønning et al. 2018). For this reason, we deemed it appropriate to include covariates at both the individual and contextual levels in our analysis.

At the individual level, we included sex (categorized as male (reference category) and female), and the annual income of each individual. We constructed this variable from two others: the proportion of patient out-of-pocket contribution and the maximum monthly out-of-pocket contribution, both for prescription cost-sharing (Moreno-Vásquez et al. 2024). We categorized the annual income of each individual as: exempted from payment (reference category); < €18,000; between €18,001 and €100,000; > €100,000; and civil servants. Exempted from payment are individuals from a lower income level, specifically those earning less than 5,635 euros, those receiving a non-contributory pension, those benefiting from the Minimum Vital Income, and the unemployed who are no longer entitled to a subsidy. Prescription cost-sharing for many civil servants is managed through a public mutual insurance company and not through the public health service, which is why it is not recorded in SIDIAP.

At the contextual level, we considered various indicators of the socioeconomic status of the basic health area (ABS, from the Spanish, *Área Básica de Salud*) where the individual resided, including the percentage of the population aged 65 years or older. Generally, a higher percentage of elderly individuals is associated with a more economically disadvantaged ABS. This variable was categorized into quartiles, with the first quartile being the reference category. We also included an indicator of the type of rurality of the ABS, categorized into rural/semi-rural/semi-urban (reference category) and urban. Rural is defined as a municipality with a population density < 100 inhabitants/km^2^ and/or less than 7,500 inhabitants. Semi-rural areas are those with 7,500 to 10,000 inhabitants and fewer than 100 inhabitants/km^2^. Areas with more than 100 inhabitants/km^2^ are considered semi-urban. Urban areas are municipalities with more than 10,000 inhabitants and more than 100 inhabitants/km^2^. The average net income per person in the ABS for the years 2015 to 2019 (INE 2025b), was categorized into quartiles, with the first quartile (representing the most economically disadvantaged group), serving as the reference category. Finally, the Gini index (INE 2025b), which measures the degree of income inequality across different regions (with a higher value indicating greater inequality), was calculated for each ABS and categorized into quartiles, with the first quartile serving as the reference category. These last two variables were calculated as a weighted average of the average net income per person and the Gini index of the census tracks that made up the ABS. The weights used were based on the population of each census tract (INE, 2025c).

As covariates used solely in the matching of cases and controls (see next section), we considered an individual-level variable—complexity indicators (complex -CCP- and advanced -MACA-)—and a contextual-level variable, which was the region where the individual resided (Lleida, Tarragona, Barcelona, Girona, the Southern Metropolitan area of Barcelona, the Northern Metropolitan area of Barcelona, Central Catalonia, the Pyrenees and the Ebre Region).

CCP is defined as an individual with multiple chronic pathologies or a single, sufficiently serious condition, often progressive, that is associated with functional, clinical, cognitive and/or social fragility. Such individuals typically require multiple resources and drugs and face a risk of iatrogenicity. It is also assumed that they could benefit from a multidisciplinary approach and comprehensive care strategies^[23]^. A MACA (chronic complex patient) is an individual whose profile is comparable to that of a CCP, but with a more severe condition and a life expectancy of less than 24 months. These individuals often require progressive, primarily palliative care, along with advanced decision planning and greater support for their caregiving structure (Gual et al. 2017).

#### Propensity scoring matching

We consider two sets of cases and controls: one corresponding to the first objective and another for the second. Matching was performed for each set. For our first objective, the sets of cases and controls were those whose marker records were available in the pre-pandemic period. For the second objective, the set of cases were centenarians for whom marker records were available in the pre-pandemic and pandemic periods, while the controls were individuals who did not achieve centenarian age in the observation period and had marker records in both pre-pandemic and pandemic periods. In both sets, each case was matched with a unique control (1:1 matching). This approach has been shown to retain statistical power, reduce variance and prevent low-quality matches (Stuart 2010).

In both matching procedures, we performed nearest neighbour matching without replacement (Ho et al. 2011), using propensity scores estimated via logistic regression. In this model, the dependent variable was the case/control indicator (used as the distance measure), and the predictors were the covariates in which we observed imbalances (i.e. explanatory variables). In the second matching, a *caliper* was applied because the balancing results after the initial matching runs were not satisfactory. A caliper sets a distance threshold for the propensity score matching (Austin 2011). A smaller caliper means that the matched individuals in each group have more similar scores. This helps ensure that the groups being compared are more similar in terms of their probabilities, thereby enhancing the validity of the study results.

In both cases, balance diagnostics were performed to assess how well the matching procedure had eliminated differences in covariate distributions between the case and control groups. We examined standardized mean differences and performed t-tests to ensure that no significant differences remained between the matched groups on the covariates. When imbalances were detected, we refined the matching process by adjusting the propensity score model, resulting in the final methods described earlier. The matching algorithms were performed using the Matchlt package (Ho et al. 2011).

### Data analysis

For each of the objectives, each categorized blood-based biomarker, and each biomarker improvement indicator, we specified a generalized linear mixed model (GLMM) with binomial link function.

$$\text{log}\left(\frac{Prob\left({centenarian}_{i}=1\right)}{1-Prob\left({centenarian}_{i}=1\right)}\right)= {\eta }_{i}+{\nu }_{k}+ S\left({ABS}_{i}\right)$$

where the subindex *i* denoted the individual; the subindex *k* the matched case–control pair; *centenarian* was an indicator of case (i.e. being a centenarian); η the linear predictor; and $\nu$ and S(ABS) denoted random effects.

We included two random effects in the models. First, ${\nu }_{k}$, random effects indexed on the matched case–control pair. These random effects were unstructured (independent and identically distributed random effects), and captured ‘individual’ heterogeneity, i.e. unobserved confounders specific to the matched pair and invariant in time.

Second, we included structured random effects to control spatial dependency, denoted as $S\left({ABS}_{i}\right)$, where ABS refers to the basic health area where the individual resided. This approach acknowledges that small areas close in space tend to show more similar values ​​of the outcome variables than geographically distant areas, reflecting unobserved contextual factors that could influence the probability of becoming a centenarian.

The spatially structured random effect S was normally distributed with a zero mean and a Matérn covariance function:

$$Cov\left(S\left({x}_{i}\right),S\left({x}_{{i}{\prime}}\right)\right)=\frac{{\sigma }^{2}}{{2}^{\nu -1}\Gamma \left(\vartheta \right)} {\left(\upkappa \Vert {x}_{i}-{x}_{{i}{\prime}}\Vert \right)}^{\vartheta } {\text{\rm K}}_{\vartheta } \left(\upkappa \Vert {x}_{i}-{x}_{{i}{\prime}}\Vert \right)$$

where ${\text{\rm K}}_{\vartheta }$ is the modified Bessel function of the second type and order $\vartheta>0$. $\vartheta$ is a smoother parameter; ${\sigma }^{2}$ is the variance; and $\kappa>0$ is related to the range ($\rho =\sqrt{8 \vartheta }/\kappa$), the distance to which the spatial correlation is close to 0.1 (Lindgren et al. 2011).

The linear predictor of the GLMM for the first objective—assessing the influence of blood-based biomarkers on the probability of becoming a centenarian—included the biomarkers (in quintiles) and all the covariates.

In the linear predictor of the GLMM for the second objective—assessing the effects of the variation in these biomarkers during the pandemic on the probability of being a centenarian—we included the indicator of improvement in the standardized variation of the indicator and, in addition to the covariates, the value of the biomarker before the pandemic.

Note that we included sex and the annual income of each individual as covariates, even though these variables were also used in the matching process. This is because we believe that the distribution of these variables between cases and controls may have varied over the study period, particularly during the COVID-19 pandemic.

#### Inference

Inferences were made following a Bayesian perspective, using the INLA approach (Rue et al. 2009 and 2014). We used priors that penalize complexity (called PC priors). These priors are robust in that they do not impact the results (Simpson et al. 2017*)*. The specifications of these priors are provided in Table S2 in the supplementary material.

All analyses were carried out using the open-source software R (version 4.4.1) (R Core Team. R 2024), through the INLA package (Rue et al. 2009 and 2014; R Inla Project 2025) in the experimental mode (Van Niekerk et al. 2022).

## Results

### Descriptive analyses

Table 1 presents the characteristics of the candidate population, defined as individuals born before 1 January 1923. Of the 30,303 individuals in the cohort who met the first two inclusion criteria, 8060 (26.60%) were either already centenarians on January 1, 2015, or they reached 100 years of age during the follow-up (2015–2022). Of these centenarians (2039 individuals) 25.3% were still alive at the end of follow-up (December 31, 2022). The median age of the centenarians on 31 December 2022 (or earlier if they had passed away) was 101 years. By that date, 10% of centenarians were 104 years or older, with one individual reaching 118 years old. Males were a minority in both groups, accounting for only 19% of the centenarians and 26.5% of the non-centenarians. Regarding annual income, 86% of centenarians earned less than €18,000 or were exempt from payment compared to 87.8% of non-centenarians. Notably, the percentage of the most economically disadvantaged individuals (those exempt from co-payment) was much higher among centenarians (24.0%) than among non-centenarians (3.8%). The percentage of individuals who earned between €18,000 and €100,000, and those who earned more than €100,000 was very similar for both centenarians and non-centenarians. Most individuals lived in urban areas, with a higher percentage of centenarians (74.9%) compared to non-centenarians (70.8%). The distribution in quartiles of the population aged 65 years or older, as well as the average net income per person, both based on the ABS where the individuals resided, were very similar between centenarians and non-centenarians. Note that in both cases, the highest percentage corresponded to the fourth quartile. In other words, they resided in ABSs with an older population and higher income. However, the distribution of the Gini index was somewhat different, with a higher percentage of centenarians residing in ABSs with greater inequality (29.3% vs 25.7% in non-centenarians).

**Table 1:** Characteristics of the subjects born before 1 January 1923

	N	Non-Centenarian (N = 22,243,73·4%)	Centenarian (N = 8,060,26·5%)
Sex	30,303		
Male		5,933 (26·7%)	1,529 (19·0%)
Female		16,310 (73·3%)	6,531 (81·0%)
Annual income individual level	28,236		
Exempted from payment		781 (3·8%)	1,795 (24·0%)
< €18,000		17,439 (84·0%)	4,630 (62·0%)
€18,000—€100,000		2.243 (10·8%)	894 (11·9%)
> €100,000		60 (0·3%)	35 (0·4%)
Civil servants		249 (1·2%)	110 (1·5%)
Population aged 65 years or older—ABS	25,872		
Q1 12·31%-17·09%		3,857 (20·9%)	1,532 (20·6%)
Q2 17.10%-19·06%		4,565 (24·8%)	1,761 (23·7%)
Q3 19·07%-21·61%		4,481 (24·3%)	1,842 (24·8%)
Q4 21·62%-28·96%		5,541 (30·0%)	2,223 (30·9%)
Rurality indicator Rural and semi-rural	19,066	4,070 (29·2%)	1,288 (25·1%)
Urban		9,868 (70·8%)	3,844 (74·9%)
Average net income per person-ABS	25,872		
Q1 €8,842.8 – €12,036.3		4,186 (22·7%)	1,608 (21·6%)
Q2 €12,036.4 – €13,495.3		3,460 (18·8%)	1,257 (16·9%)
Q3 €13,495.4 – €14,855.2		4,638 (25·1%)	1,836 (24·7%)
Q4 €14,855.3 – €24,682.3		6,160 (33·4%)	2,727 (36·7%)
Gini index—ABS	25,872		
Q1 13·25%-28·54%		3,877 (21·0%)	1,486 (20·0%)
Q2 28.55%-30·01%		4,831 (26·2%)	1,809 (24·4%)
Q3 30·02%-31·75%		4,987 (27·0%)	1,954 (26·3%)
Q4 31·76%-39·71%		4,749 (25·7%)	2,179 (29·3%)
Region	30,303		
Lleida		1,671 (7·5%)	596 (7·4%)
Tarragona		1,143 (5·1%)	486 (6·0%)
Barcelona		5,397 (24·3%)	2,200 (27·3%)
Girona		2,412 (10·8%)	765 (9·5%)
Metropolitan South		3,602 (16·2%)	1,311 (16·3%)
Metropolitan Nord		4,700 (21·1%)	1,599 (19·8%)
Central Catalonia		2,032 (9·1%)	663 (8·2%)
High Pyrenees		317 (1·4%)	112 (1·4%)
Ebro Lands		969 (4·4%)	328 (4·1%)
Complexity status	30,303		
Chronic Patient – CCP		7,023 (31·6%)	2,657 (33·0%)
Advanced chronic disease—MACA		5,091 (22·9%)	1,996 (24·8%)
No information		10,129 (45·5%)	3,407 (42·3%)

N is the number of non-missing values

The complexity of centenarians and non-centenarians was very similar, about one-third were complex patients (CPP) and approximately one-fifth were classified as advanced chronic patients (MACA). As regards the region where the individuals resided, most of both centenarians and non-centenarians lived in Barcelona and its metropolitan regions (63% aggregated). Girona accounted for 9.5%, while Tarragona, Central Catalonia, and Lleida were evenly distributed (around 7% each).

The results of applying the third inclusion criteria—at least one blood test registered in the SIDIAP database—reduced the candidate pool to 25,872 individuals (85.38% of the candidate population): 7,428 centenarians (93.16%), and 18,444 non-centenarians (82.92%) (see Table S3 in the supplementary material).

Table 2 shows the descriptives of the blood-based biomarkers measured in the last blood test taken before April 2020. The mean (and the median) levels of iron, haemoglobin (anaemia biomarkers), all the cholesterol biomarkers (total cholesterol, HDL-C and LDL-C) and CKD-EPI (kidney function biomarker) were higher in centenarians than in non-centenarians. In contrast, ferritin (anaemia biomarker), fasting blood glucose (glycemia biomarker), urea and creatinine (kidney function biomarkers), and ALP (a liver function biomarker) were higher in non-centenarians. Although the median of the HbA1c levels was the same in centenarians and non-centenarians, note that levels in the upper quintiles (60% and 80%) were higher in non-centenarians than in centenarians.

**Table 2:** Descriptive of blood-based biomarkers measured in the last blood test taken before April 2020^a^

Biomarker	Non-centenarians	Centenarians
Anaemia biomarkers		
Iron	56.23 (27.18) [33.0,48.0,**54.0**,61.0,75.2]	61.29 (25.63) [39.1,54.0,**58.9**,66.0,81.0]
Haemoglobin	11.46 (1.92) [9.9,11.1,**11.6**,11.9,13.1]	11.80 (1.69) [10.6,11.5,**11.9**,12.3,13.1]
Ferritin	177.20 (230.13) [41.0,78.8,**107.0**,141.0,256.4]	149.51 (176.50) [38.2,70.1,**94.2**,117.4,218.9]
Cholesterol biomarkers		
Total cholesterol	166.47 (44.67) [131.0,154.0,**164.0**,175.0,201.6]	175.22 (42.54) [139.0,163.0,**172.0**,182.0,206.0]
HDL-C	46.84 (13.18) [36.0,42.0,**45.0**,49.0,57.0]	49.57 (13.43) [38.0,45.0,**48.0**,51.0,49.0]
LDL-C	98.44 (33.98) [72.0,88.0,**96.0**,104.0,127.0]	103.97 (33.95) [76.0,94.0,**101.0**,109.0,130.0]
Glycemia biomarkers		
HbA1c	6.26 (1.19) [5.4,5.7,**5.9**,6.2,7.0]	6.08 (0.90) [5.4,5.7,**5.9**,6.0,6.6]
Fasting blood glucose	115.49 (54.14) [82.0,92.0,**99.0**,109.0,142.0]	106.05 (39.60) [80.0,89.0,**94.0**,100.0,125.0]
Kidney function biomarkers		
CKD-EPI	45.51 (19.89) [27.3,38.2,**43.4**,49.6,66.7]	48.23 (18.25) [31.5,42.5,**47.7**,52.9,66.2]
Urea	69.76 (45.24) [37.5,51.0,**59.0**,66.0,94.0]	61.31 (32.71) [37.0,50.0,**55.0**,61.0,78.0]
Creatinine	1.34 (0.73) [0.80,1.03,**1.15**,1.30,1.70]	1.17 (0.55) [0.77,0.95,**1.05**,1.15,1.48]
Liver functioning biomarker		
ALP	103.70 (61.53) [67.0,83.0,**90.0**,100.0,126.0]	100.22 (59.45) 68.0,80.0,**85.0**,94.0,121.0]

*HDL-C* high-density lipoprotein cholesterol, *LDL-C* low-density lipoprotein cholesterol, *HbA1c* glycosylated haemoglobin, *CKD-EPI* glomerular filtration rate, *ALP* alkaline phosphatase

^a^First row: Mean (standard deviation)

Second row: [first quintile, second quintile, **median**, third quintile, fourth quintile]

Table 3 shows the most prevalent chronic conditions at baseline. While 92.15% of centenarians (7,468 of 8,060) suffered from one of the most prevalent chronic conditions, compared with 82.54% of non-centenarians (18,444 of 22,343). The most prevalent diagnoses in both centenarians and non-centenarians were, in descending order, disease of the circulatory system (heart failure, cardiac dysrhythmias, and hypertension); and endocrine, nutritional and metabolic diseases. Within this group, the most prevalent chronic conditions were nutritional deficiencies. The order of conditions then varied: among centenarians the most prevalent were thyroid disorders, obesity and type 2 diabetes mellitus (in descending order), while among non-centenarians these three chronic conditions had very similar prevalences. Unlike non-centenarians, centenarians had the lowest prevalence of neurocognitive and mental disorders.

**Table 3:** Most prevalent comorbidities at baseline

	Non-Centenarian	Centenarian
	(N = 18,444, 82·54%^a^)	(N = 7428, 92·15%^a^)
Diseases of the blood		
Aplastic Anaemia	2102 (11·4%)	1208 (16·3%)
Diseases of the circulatory system		
Essential hypertension	1475 (8·0%)	840 (11·3%)
Cardiac dysrhythmias	2024 (11·0%)	979 (13·2%)
Heart failure	2208 (12·0%)	1035 (13·9%)
Endocrine, nutritional and metabolic diseases		
Type 2 diabetes mellitus	644 (3·5%)	285 (3·8%)
Nutritional deficiencies	1496 (8·1%)	1118 (15·1%)
Thyroid disorders	600 (3·3%)	391 (5·3%)
Obesity	577 (3·1%)	296 (4·0%)
Diseases of the genitourinary system		
Chronic kidney disease	2072 (11·2%)	1309 (17·6%)
Mental disorders		
Depressive disorders	931 (5·0%)	447 (6·0%)
Anxiety and fear-related disorders	983 (5·3%)	609 (8·2%)
Tobacco-related disorders	405 (2·2%)	105 (1·4%)
Diseases of the nervous system		
Neurocognitive disorders	2454 (13·3%)	1198 (16·1%)

Percentage of the total number of non-centenarians and centenarians

Table S4 in the supplementary material shows the centenarians and non-centenarians who experienced an improvement in the standardized variation of the biomarkers during the pandemic compared to the period before it. As shown, the percentages of individuals who improved were very similar between the two groups for most biomarkers. Somewhat higher was the improvement for non-centenarians in haemoglobin, ferritin, LDL cholesterol, fasting blood glucose, and urea, while it was somewhat lower for, iron, total cholesterol, HDL-C cholesterol, glomerular filtration rate (CKD-EPI), creatinine and alkaline phosphatase (ALP). However, for glycosylated haemoglobin (Hb1Ac), the percentage of non-centenarians who improved was considerably higher than in centenarians (around 5% more).

### Propensity score matching results

#### Matching for the pre-pandemic period centenarians

The cases consisted of 3,890 individuals who achieved centenarian status in the pre-pandemic period, lived in Catalonia before the pandemic began, and had measurements of at least one marker in that period but were not present in the pandemic period due to transfer, death or lack of marker records. The controls were 17,307 individuals who died during the same pre-pandemic period and had biomarkers recorded.

After screening all covariates, we found imbalances between cases and controls for sex, annual income of each individual, region and complexity. The covariates used for matching were sex, annual income, region, and complexity. The balancing was assessed and deemed satisfactory given that the values of standardized mean differences were close to zero, and values of variance ratios were close to 1. In this case, the matching was complete (see Table S5 and Figure S1 in the supplementary material).

#### Matching for the centenarians active in the COVID-19 pandemic period

For this analysis, the cases consisted of 3,538 individuals who achieved centenarian status in the pre-pandemic or pandemic periods and had records of biomarkers within this time frame. The controls were all 18,444 non-centenarians in the sample (see Table S5 in the supplementary material).

Covariates with imbalances were sex, annual income, and complexity. An initial 1:1 nearest neighbour propensity score matching without caliper restrictions yielded complete matching across all cases. However, the assessment of covariate balance, using standardized mean differences and variance ratios, revealed persistent bias in the annual income variable (see Figure S2 in the supplementary material). Alternative matching methods, including optimal and genetic algorithms, did not lead to any improvements regarding this imbalance. To address this, a caliper restriction on the propensity score distance was applied to enhance the quality of the match. This approach substantially reduced bias in annual income but, as a side effect, also resulted in the exclusion of cases without a sufficiently close match. Specifically, 561 individuals (15.8% of the original treatment group) were unmatched. These unmatched individuals were predominantly women (95%), categorized as ‘exempt from payment’ in annual income, and had a complex status (CPP) recorded. After applying the caliper, balance diagnostics showed notable improvements across all covariates, confirming enhanced match quality despite the reduced sample size.

### Modelling results

**Fig. 1:**
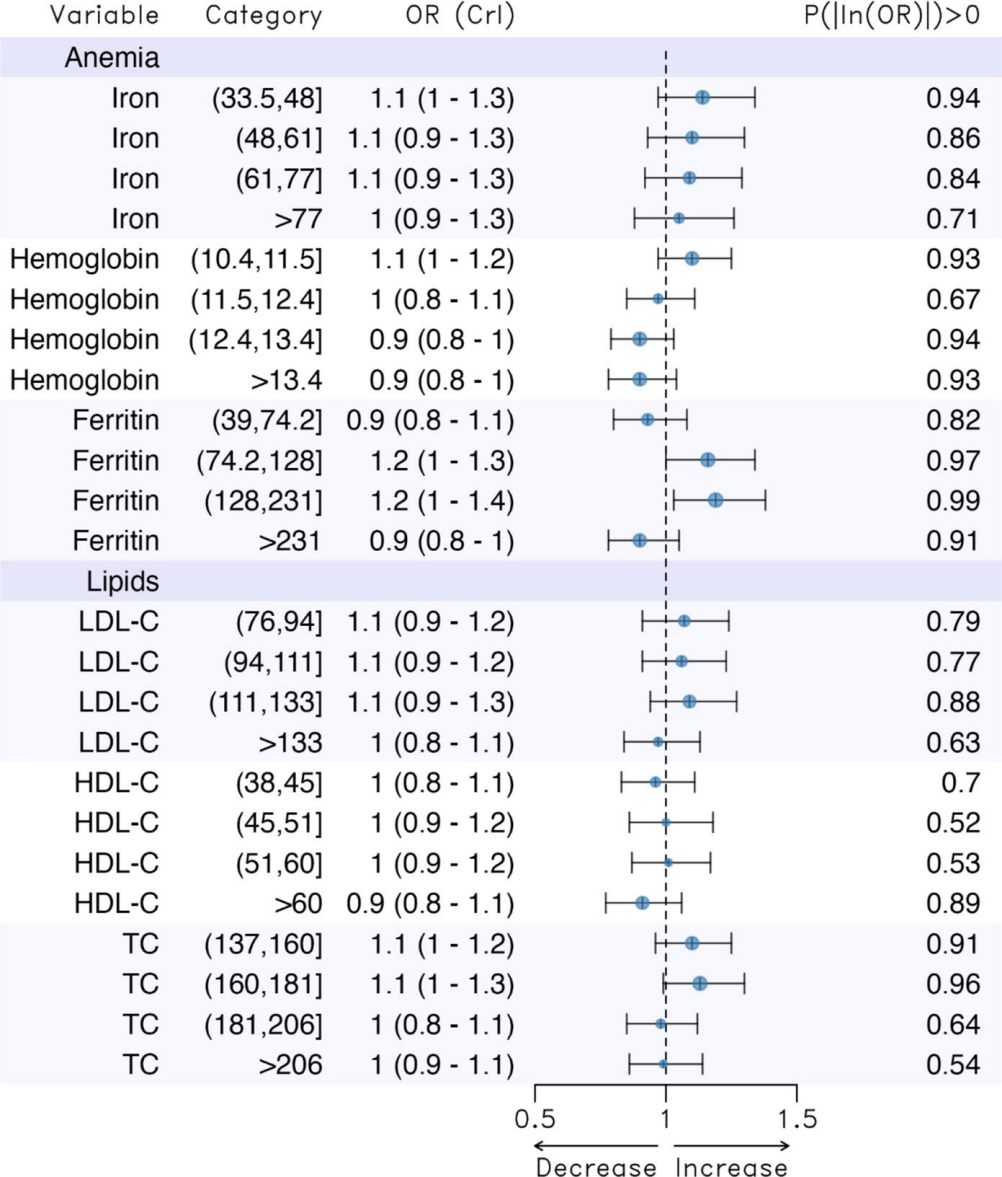
Results of the models relating blood-based biomarkers and the probability of being a centenarian. *LDL-C* low-density lipoprotein cholesterol, *HDL-C* high-density lipoprotein cholesterol, *TC* Total cholesterol. Adjusted for sex, annual income of each subject (annual income), percentage of the population aged 65 years or older (Population_65_or_more), rurality, average net income per person (contextual income) and the Gini index. *OR* odds ratio, *CrI* 95% credibility interval, *P(|log(OR|)* > 0 probability that the coefficient (log(OR)) is not equal to zero

**Fig. 2:**
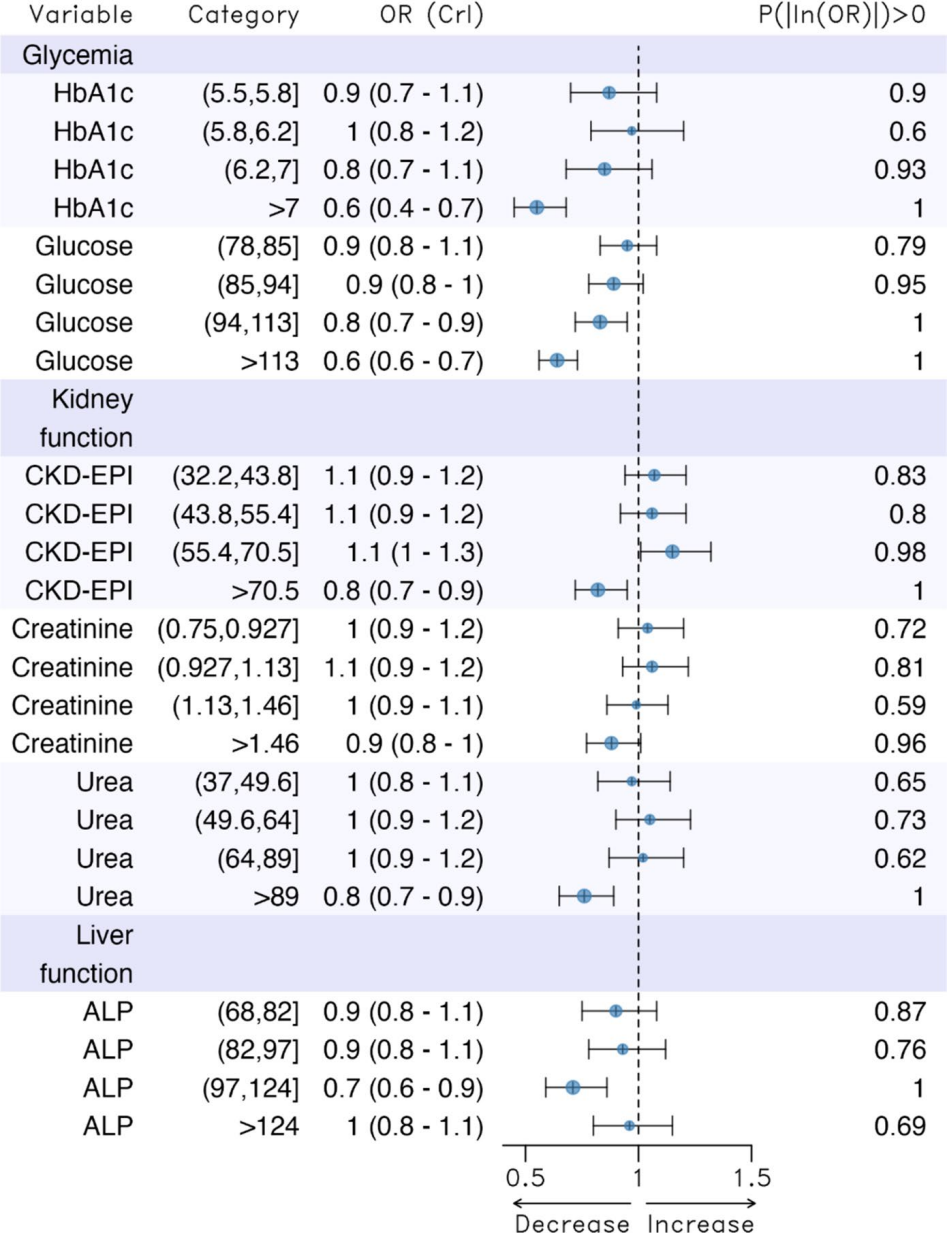
Results of the models relating blood-based biomarkers and the probability of being a centenarian. *HbA1c* glycosylated haemoglobin, *Glucose* Fasting blood glucose, *CKD-EPI* glomerular filtration rate, *ALP* alkaline phosphatase. Adjusted for sex, annual income of each subject (annual income), percentage of the population aged 65 years or older (Population_65_or_more), rurality, average net income per person (contextual income) and the Gini index. *OR* odds ratio, *CrI* 95% credibility interval, *P(|log(OR|)* > 0 probability that the coefficient (log(OR)) is not equal to zero

The results of the models relating blood-based biomarkers and the probability of being a centenarian are categorized by groups of biomarkers and displayed in Figs. 1, 2. The figures provide the ORs, their 95% credible intervals (equivalent to 95% confidence intervals), and the probability of the logarithm of the OR being greater than zero (i.e. the probability that the OR was not equal to 1). In general, when this probability is greater than (or equal to) 0.95, the 95% credibility interval of the OR will not contain the unit.

We estimated ORs for which the 95% credibility intervals did not contain unity in ferritin (third and fourth quintiles), total cholesterol (second quintile), HbA1c (fifth quintile), fasting blood glucose (fourth and fifth quintiles), CKD-EPI (fourth and fifth quintiles), urea (fifth quintile), and ALP (fourth quintile).

Except for ferritin, total cholesterol and the fourth quintile of CKD-EPI, compared to the lowest levels (first quintile), having the highest levels (fourth and fifth quintiles) of these biomarkers, as well as the second quintile for total cholesterol, was associated with a lower propensity to be a centenarian. This reduction ranged from 20% less (in CKD-EPI and urea) to 40% less (in HbA1c and fasting blood glucose), with a 30% reduction in ALP. In contrast, higher levels of ferritin, the second quintile of total cholesterol, and the fifth quintile of CKD-EPI were associated with a greater propensity to become a centenarian—around 20% more for ferritin and 10% more for total cholesterol and CKD-EPI—compared to the reference category (the lowest levels).

**Fig. 3:**
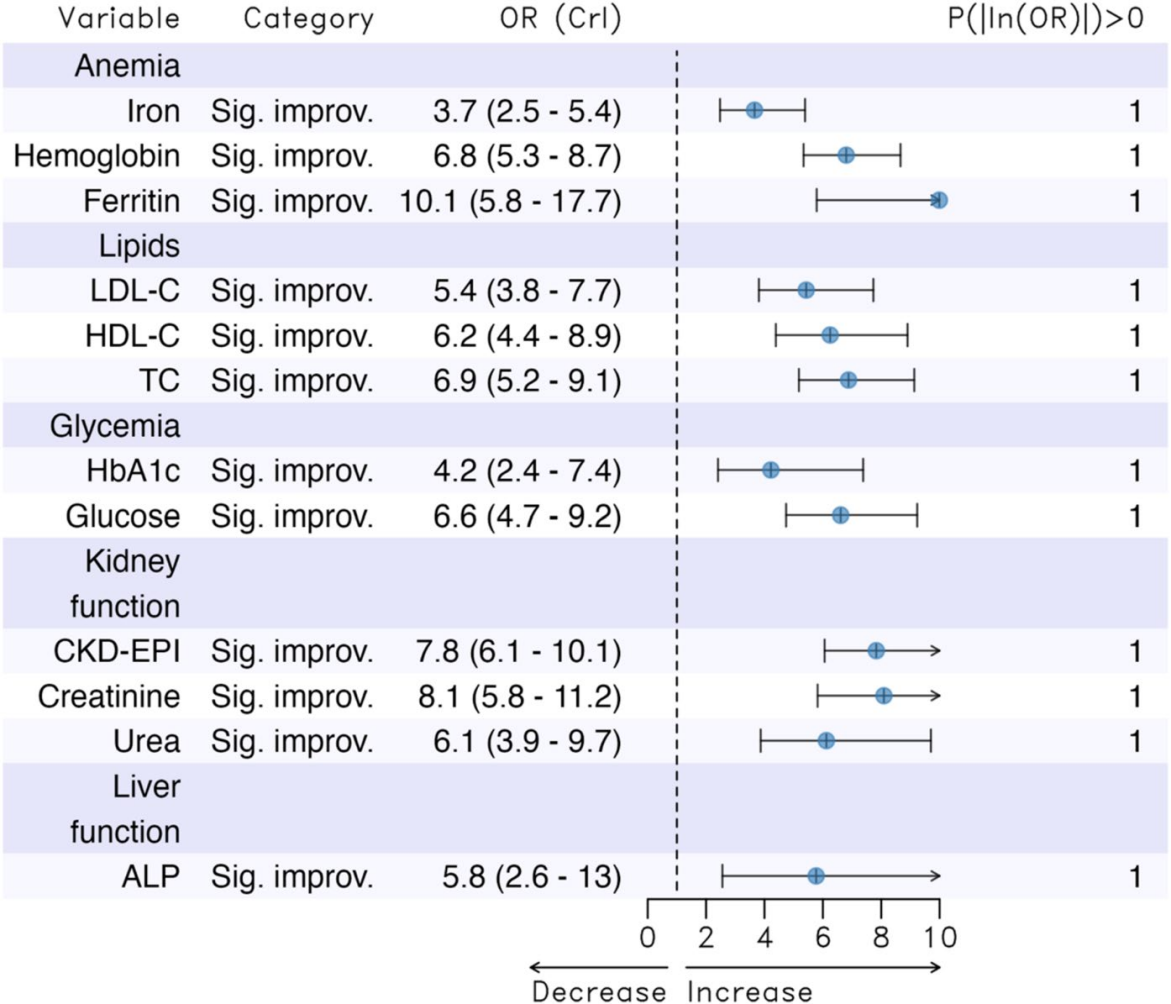
Results of assessing how variations in the biomarkers during the COVID-19 pandemic influenced the probability of attaining centenarian age. *LDL-C* low-density lipoprotein cholesterol, *HDL-C* high-density lipoprotein cholesterol, *TC* Total cholesterol, *HbA1c* glycosylated haemoglobin, *Glucose* Fasting blood glucose, *CKD-EPI* glomerular filtration rate, *ALP* alkaline phosphatase. Adjusted for sex, annual income of each subject (annual income), percentage of the population aged 65 years or older (Population_65_or_more), rurality, average net income per person (contextual income), the Gini index and the level of each biomarker before the pandemic (April 2020). *OR* odds ratio; *CrI* 95% credibility interval, *P(|log(OR|*) *> *0: probability that the coefficient (log(OR)) is not equal to zero

The results of assessing how variations in the biomarkers during the COVID-19 pandemic influenced the probability of attaining centenarian age are shown, again grouped by biomarkers, in Fig. 3. As observed, in all cases the 95% OR credibility intervals did not contain unity. Furthermore, we found that an improvement (in the standardized variation of the indicator) in all biomarkers during the pandemic (compared to no variation or worsening) increased the likelihood of becoming a centenarian.

## Discussion

In our cohort, we observed that, despite being a minority in both groups, centenarians had a lower proportion of men compared to non-centenarians (19% vs 26.7%, respectively). Additionally, the percentage of centenarians in the most economically disadvantaged category was six times higher than that of non-centenarians (24.0% vs 3.8%, respectively). A higher percentage of centenarians also lived in urban areas (74.9% vs 70.8% in non-centenarians). Moreover, although centenarians resided in ABSs with similar age distributions and income levels as non-centenarians, a higher percentage of them lived in ABSs with the greatest inequality (29.3% vs 25.7% in non-centenarians).

On the other hand, centenarians had a higher prevalence of the most common chronic conditions compared to non-centenarians (92.15% vs 82.54% respectively). Except for type 2 diabetes mellitus (which had similar prevalence rates for both groups) and tobacco-related disorders (which were more prevalent among non-centenarians), the baseline prevalence of chronic conditions was higher among centenarians than among non-centenarians.

Regarding our first objective, our findings align with previous studies that underline the important role blood biomarkers play in longevity, while also introducing key nuances. Like Murata et al. (2024), we observed that elevated levels of ferritin and total cholesterol in certain ranges (intermediate quartiles, specifically the second and third in our case), together with low levels of glucose, creatinine and uric acid, are associated with a higher probability of reaching 100 years of age.

It should be noted that, in our case, high total cholesterol levels (fourth and fifth quintiles) were not associated with the propensity to be a centenarian. Blaton, however, points out that elevated cholesterol, often linked to metabolic syndrome, has been found to be associated with an increased risk of cardiovascular events (Blaton 2007). Nevertheless, the role cholesterol plays in longevity is not at all clear. In this sense, Ravnskov et al. (2016), in a systematic review of 19 cohort studies, including 30 cohorts with a total of 68,094 individuals ≥ 60 years from the general population, found that high LDL cholesterol is inversely associated with mortality in most people over 60 years. Similarly, Wang et al. (2023), in a prospective cohort based on the Beijing Elderly Comprehensive Health Cohort Study, which included 4,499 community-dwelling older adults (aged ≥ 60 years), found that higher levels of total cholesterol and HDL cholesterol were associated with lower cardiovascular mortality in normal lipid reference range. They proposed remnant cholesterol as a more reliable lipid indicator for estimating cardiovascular death risk in older adults (Wang et al. 2023). Barzilai et al. (2003) examined the lipoprotein phenotype and genotype in long-lived individuals of Ashkenazi Jewish origin, finding significantly larger HDL and LDL particle sizes in the long-lived individuals and their offspring compared to controls. This lipoprotein phenotype was associated with a lower prevalence of hypertension, cardiovascular diseases, and metabolic syndrome. Unlike them, we did not find statistically significant differences in LDL and HDL cholesterol levels when assessing the likelihood of reaching centenarian age. The lack of statistical significance for LDL and HDL cholesterol, in contrast to intermediate total cholesterol levels, could be explained by findings from Barcilai et al*.* (2023). Their study shows that, compared to non-centenarians, centenarians have differential qualitative patterns in HDL and LDL (e.g. larger LDL particle size), but this does not necessarily mean that they have differentiated HDL and LDL levels. Indeed, Atzmon et al. (2006) show that the lipid profile of centenarians is characterized more by qualitative patterns (for instance, cholesterol ester transfer protein gene activity), than by isolated LDL/HDL levels.

The fact that biomarkers related to glucose metabolism, such as HbA1c and fasting glucose, have a significant negative impact on the probability of becoming a centenarian when their values ​​are in the highest quintiles, complements the observations of Ding et al. (2021), who highlighted the importance of maintaining a lipid and glycaemic balance in cardiovascular mortality at advanced ages. Unlike Murata et al. (2024), we did find that elevated levels of alkaline phosphatase, as well as CKD-EPI, were also associated with a lower propensity of becoming a centenarian.

Biomarkers related to anaemia, haemoglobin and serum iron are tightly regulated by homeostatic mechanisms (for instance, hepcidin), which may limit their variability in older adults and reduce their ability to show associations with extreme longevity. In contrast, ferritin reflects long-term iron storage (Pietrangelo 2016), and is also modulated by inflammation and oxidative stress, making it a more sensitive marker of cumulative iron-related damage (Kell and Pretorius 2014). Clinical studies have reported that elevated ferritin (but not serum iron) predicts mortality in aging populations, independent of haemoglobin levels (Mainous et al. 2004). This is consistent with our findings, where ferritin, but not iron or haemoglobin, was significantly associated with extreme longevity.

However, regarding our second objective, we found that an improvement in the standardized variation of all the biomarker indicators during the pandemic (compared to no variation or worsening) was associated with an increased likelihood of reaching centenarian age. These findings were found to be not sensitive to the selection of the improvement cut-off points at different percentiles (Table S7 and Figure S3 in the supplementary material), with the only exception being the Alkaline phosphatase biomarker (Figure S3 in the supplementary material), whose significance is diluted when applying a strict 5th percentile. Other biomarker parameters increased the width of the credibility intervals, but their significance remained high without including 1 and a smaller sample size.

While several studies report increased metabolic risks during the pandemic (especially rising BMI) (Chiavarini et al. 2024; Moreno-Vásquez et al*.*, 2024), our findings suggest that individuals with improved biomarker stability were more likely to achieve extreme longevity. The pandemic may thus have acted as a selective stressor, revealing a subset with inherent robustness, possibly due to genetics, lifestyle or healthcare-access advantages.

As is well known, an increase in ‘healthy life expectancy’ is just as important, if not more so, than an increase in overall life expectancy. Coined by the WHO, this term refers to the average number of years that a person can expect to live in ‘full health’, omitting the years spent in less healthy conditions due to illness or injury (World Health Organization 2025). In this context, there is some controversy over whether centenarians can truly be considered to have experienced ‘successful ageing’. Rowe and Kahm (1997) defined successful ageing as the absence of disease and disability, maintenance of high levels of physical and cognitive abilities, and preservation of social and productive activities. Motta et al. (2005) concluded that centenarians did not represent the prototype of successful ageing. They suggest that if negative environmental factors could be overcome in the future, it might not only be possible to increase the average life expectancy of genetically predisposed subjects, but also to maintain a high quality of life and thus, this would extend the concept of real successful ageing to centenarians as well. Nevertheless, Fouweather (2005) observed that centenarians had fewer comorbidities and hospitalisations and better cognitive function than non-centenarians, so they could be considered model cases of ‘successful ageing’. Musich et al. (2022) define resilience as the ability to recover from disturbances caused by stressors such as illness, falls or hospitalizations due to diseases such as cancer. Recent studies have brought resilience to the forefront of ageing research, highlighting its role in mitigating age-related challenges (Merchant et al. 2022). In line with this, Islam et al. (2024)*,* highlight that centenarians possess genetic, physical, and cognitive capabilities that enable them to counteract adverse influencing factors, thereby allowing them to live longer than the average human. According to their findings, centenarians attribute their long and healthy lifespan to a combination of genetic predispositions and favourable environmental factors. Notably, these resilient individuals show impressive resistance to common morbidity factors such as cardiovascular disease, cancer, neurodegenerative diseases, diabetes, and renal disorders.

Furthermore, analysing demographic survival metrics from national vital statistics in the eight countries with the longest-lived populations (Australia, France, Italy, Japan, South Korea, Spain, Sweden, and Switzerland) and in Hong Kong and the USA from 1990 to 2019, Olshansky et al. (2024) noted that since 1990, overall improvements in life expectancy have slowed. Their analysis also revealed that resistance to improvements in life expectancy increased, while inequality in life expectancy decreased, and mortality was compressed. As far as centenarians are concerned, 2010 appears to mark a turning point. Children born after that year have a lower probability of reaching 100 than had been projected previously, had the rate of increase continued. Specifically, 5.3% probability for women and 1.8% probability for men- figures lower than those suggested by earlier projections. By region, the highest country-specific probability of children born in 2019 surviving to age 100 is found in Hong Kong, where 12.8% of women and 4.4% of men are expected to reach 100. In contrast, the United States has a much lower expectation, with only 3.1% of women and 1.3% of men projected to live to 100. In Spain, the estimated survival rate for 100 years in 2019 was 4.4% for women, 1.6% for men and 3.1% for the entire population. While these are higher figures than those from 2010, they are not as high as would have been expected if the same progression had been followed (Olshansky et al. 2024).

The ongoing debate around successful ageing, along with the stagnation in life expectancy improvements, shows that there are many more factors—beyond blood-based biomarkers or the chronic diseases to which they may be linked—that can influence the probability of being a centenarian. These factors occur mainly at the individual level, such as lifestyle choices (Stathakos et al. 2005; Mattson et al. 2017; Di Francesco et al. 2018), including nutrition, smoking and physical activity (Crimmings et al. 2019; Islam et al. 2024), as well as physiological factors (Kennedy et al. 2014; Epel and Lithgow 2014; Conte et al. 2019), cognitive performance (Voelcker-Rehage 2011; Oltmanns et al. 2017), exposure to prior and ongoing medical treatment (Meinow et al. 2010; Smith et al. 2011) and access to health care (Crimmings et al. 2019; Islam et al. 2024)^[2,47]^. Other influences include genetic predisposition (Sebastiani et al. 2010; Islam et al. 2024), mental health (Grønning et al. 2018), particularly anxiety and depression, education, socioeconomic level, and social support or, its opposite, social isolation (Naito et al. 2023). Additionally, contextual factors such as occupational (Crimmings et al. 2019; Islam et al. 2024) or environmental exposures (Rowe and Kahm 1997; Calabrese et al. 2015) (mainly air pollution), and even the socioeconomic level of the area where the individual lives, are also important.

This leads to the first limitation of our article. As is known, the main problem of retrospective cohort designs, such as the one we used, is the lack of control for confounding. We followed two strategies to control for this. Firstly, matching. The purpose behind employing matching is to mitigate the confounding effects that could otherwise distort the relationship between the factors of interest and longevity. For instance, without matching, individuals from wealthier backgrounds may appear more likely to become centenarians, but this could simply reflect broader access to healthcare and better living conditions rather than any inherent effect of their wealth itself. By matching individuals with similar socioeconomic profiles, we were able to isolate the impact of other variables, such as lifestyle choices or genetic factors, on longevity. This helps disentangle the complex web of interactions between variables and allows for a clearer understanding of the specific factors that are most influential in predicting the likelihood of becoming a centenarian. Secondly, we included in the multivariate models’ random effects that controlled for unobserved confounding, both at the individual level (matched case–control pair) and at the contextual level (spatial dependence). However, we do not rule out the existence of residual confounding, so our results should be interpreted with some caution.

The second limitation is the existence of selection bias. We used a primary care database (SIDIAP) in which blood tests of hospitalized individuals were not collected. Fortunately, it is unlikely that individuals were hospitalized for all or most of the study period (2015–2022), so their blood tests would also be available in primary care databases. Nonetheless, there may have been a few controls with very serious illnesses who were hospitalized for most of the study period, resulting in incomplete test data for them. This, however, would likely lead to an underestimation of the probability of becoming a centenarian.

In addition, with respect to our second objective, we acknowledge the possibility of selection bias, which may have reduced the generality of our study. Thus, in the first instance, unlike in the pre-pandemic scenario, it was not possible to match all the cases. The unmatched group consisted of 561 individuals in the category of ‘exempted from payment’ in the annual income (i.e. those with the lowest socioeconomic level), with a complex status (CPP), and 95% of them were women. Second, while only 11.20% of non-centenarians died during the COVID-19 pandemic (after April 2020), 47.75% of the centenarians passed away. Finally, the centenarians who died during the pandemic had lower levels of total cholesterol, fasting blood glucose, and urea biomarkers, higher levels of iron and ferritin, and similar values ​​for haemoglobin, HbA1c, CKD-EPI, and creatinine (Table S6 in the supplementary material).

Fourthly, although both Ahadi et al. (2020) and Murata et al. (2024) point out that immunity is crucial in the ageing process, we did not have any information on immunity-related biomarkers. A further notable limitation is the absence of data on medication use (e.g. statins or antidiabetics), which could confound the biomarker-longevity associations. Nor did we have information on individuals’ lifestyle choices (smoking, alcohol, physical exercise, etc.). Fortunately, all these misclassification biases are non-differential.

Finally, our cohort included individuals aged 92 years or older. As Murata et al. (2024) point out, health selection may begin much earlier. However, choosing older controls reduces differences in age-related risk factors and longevity. If the control group included much younger people, it would be more difficult to discern whether biomarkers associated with longevity are specific to those who reach 100 years of age or simply correlated with living to older ages in general. Having a control group close in age helps minimize this bias and provides a more direct comparison. In addition, people who live to age 90 or older have overcome many more mortality risk factors than younger people would have. If much younger controls are selected, they could include individuals who have not yet developed certain conditions or who have had less time to accumulate health risks. Older controls allow for a better study of the factors that affect longevity at the end of the age spectrum. In this sense, Vetrano et al. (2021) mention that centenarians show fewer changes in biomarkers related to inflammation and liver function compared to elderly individuals who did not reach 100 years of age. These results could indicate that specific factors related to long-term health can differentiate centenarians from those who, although long-lived, do not reach the 100-year threshold, thus justifying the use of an elderly control group for a more adjusted analysis of extreme longevity.

We believe that these limitations were offset by the strengths of this article. The sub cohort we used was drawn from a very large population-based cohort, which represented 81.6% of the population of Catalonia, and therefore, highly representative. We used matching and adjusted for a large number of confounders, both observed and unobserved. The latter allowed us to control not only for individual heterogeneity, but also for contextual geographic variability.

## Conclusions

Our study provides valuable insights into the distinct demographic, socioeconomic, and biomarker profiles of centenarians compared to non-centenarians. We observed that centenarians are characterized by higher rates of chronic conditions, a greater prevalence of socioeconomic disadvantage, and a stronger association with urban living and higher levels of neighbourhood inequality. These findings underline the interplay between individual and contextual factors that contribute to longevity.

From a biological perspective, we identified that intermediate levels of certain biomarkers, such as ferritin and cholesterol, along with low levels of glucose, creatinine, and uric acid, are associated with a higher likelihood of reaching 100 years. Notably, elevated cholesterol levels beyond a certain threshold did not correlate positively with longevity, contrasting with some prior findings. Our results also highlight the significance of glycaemic balance and suggest that markers such as HbA1c and fasting glucose play a critical role in survival to extreme old age. Additionally, improvements in biomarker levels during the pandemic were associated with a higher propensity to being a centenarian, despite potential selection biases in this cohort.

Beyond biomarkers, our findings align with existing evidence that suggests successful ageing and longevity are multifaceted phenomena. The role of resilience, as seen in centenarians’ ability to withstand chronic conditions and environmental stressors, emerges as a critical determinant. The ongoing debate over ‘successful ageing’ emphasizes the need for a broader perspective that includes genetic predispositions, lifestyle factors, mental health, and social determinants.

Ultimately, while biomarkers offer valuable insights, our findings reaffirm that longevity and healthy aging are influenced by a complex interplay of biological, behavioural, and contextual factors. Future research should aim to integrate these dimensions to further understand the pathways to longevity and to promote health span alongside lifespan.

Understanding the factors contributing to the rise of centenarians is crucial for public health planning and providing insights into aging, population sustainability, and the future social and economic implications of an aging society. Such knowledge will offer insights not only for regional policymakers, but also for global longevity research. The findings could contribute to promoting healthy aging, improving quality of life, and better planning for the needs of an increasingly aging population. By integrating the effects of temporal variations and public health context on key biomarkers, our results offer a broader understanding of longevity and its determinants.

## Supplementary Information

Below is the link to the electronic supplementary material.

Supplementary file1 (DOCX 768 KB)

## Data Availability

In accordance with the protocol approved by the Ethics Committees of Fundació Institut Universitari per a la Investigació en Atenció Primària de Salut Jordi Gol i Gurina and legal regulations (both the provisions of the Spanish Law 3/2018, of December 5, on Personal Data Protection and Guarantee of Digital Rights, such as the Collaboration Agreement for which the project SLT/3896/2021 is executed), there are restrictions on the transfer of data to third parties and data are not publicly available. The datasets (properly anonymised) used and analysed during the current study will be available from the corresponding author on reasonable request. Codes will be available at www.researchprojects.es. Study documents, including the protocol, will be made available upon request from the corresponding author.
