# Supplementary material for: Blood-based biomarkers in centenarians and non-centenarians: a matched, population-based retrospective cohort study using primary care records in Catalonia, Spain
Source: Biogerontology. 2025 May 26;26(3):115. doi: 10.1007/s10522-025-10258-3 (PMC12106579; doi:10.1007/s10522-025-10258-3)

Table S1. RECORD checklist^1^

| **Section and Topic** | **Item #** | **STROBE items** | **Location in**  **manuscript where**  **items are reported** | **RECORD items** | **Location in**  **manuscript**  **where items are**  **reported** |
| --- | --- | --- | --- | --- | --- |
| **TITLE AND ABSTRACT** | | |  |  |  |
| Title | 1 | (a) Indicate the study’s design  with a commonly used term in  the title or the abstract (b)  Provide in the abstract an  informative and balanced  summary of what was done and  what was found | Page 1; Title page  Page 2: Abstract  Page 1; Title page | RECORD 1.1: The type of data used should be specified in the title or abstract. When possible, the name of the databases used should be included.  RECORD 1.2: If applicable, the geographic region and timeframe within which the study took place should be reported in the title or abstract.  RECORD 1.3: If linkage between databases was conducted for the study, this should be clearly stated in the title or abstract. | Page 1; Title page  Page 2: Abstract  Page 1; Title page |
| **INTRODUCTION** | | |  |  |  |
| Background  rationale | 2 | Explain the scientific  background and rationale for the  investigation being reported | Pages 3-4; Introduction |  | Pages 3-4; Introduction |
| Objectives | 3 | State specific objectives,  including any prespecified  hypotheses | Page 4; Introduction |  | Page 4; Introduction |
| **METHODS** | | |  |  |  |
| Study Design | 4 | Present key elements of study  design early in the paper | Page 4; Methods, Study Design, Data Source, and Population |  | Page 4; Methods, Study Design, Data Source, and Population |
| Setting | 5 | Describe the setting, locations,  and relevant dates, including  periods of recruitment, exposure,  follow-up, and data collection | Pages 4-5; Methods, Study Design, Data Source, and Population |  | Pages 4-5; Methods, Study Design, Data Source, and Population |
| Participants | 6 | (a) Cohort study  - Give the  eligibility criteria, and the  sources and methods of selection  of participants. Describe  methods of follow-up.  Case-control study  - Give the eligibility criteria, and the sources and methods of case  ascertainment and control  selection. Give the rationale for  the choice of cases and controls  Cross-sectional study  - Give the eligibility criteria, and the sources and methods of selection of participants  (b) Cohort study  - For matched  studies, give matching criteria  and number of exposed and  unexposed  Case-control study  - For matched studies, give matching criteria and the number of controls per case | Pages 4-5; Methods, Study Design, Data Source, and Population.  Table S2.  Reference 15, describes the same database (SIDIAP):  Not applicable | RECORD 6.1: The methods of study population selection (such as codes or algorithms used to identify subjects) should be listed in detail. If this is not possible, an explanation should be provided.  RECORD 6.2: Any validation studies of the codes or algorithms used to select the population should be referenced. If validation was conducted for this study and not published elsewhere, detailed methods and results should be provided.  RECORD 6.3: If the study involved linkage of databases, consider use of a flow diagram or other graphical display to demonstrate the data linkage process, including the number of individuals with linked data at each stage. | Pages 4-5; Methods, Study Design, Data Source, and Population.  Table S2.  Reference 15, describes the same database (SIDIAP):  Not applicable |
| Variables | 7 | Clearly define all outcomes,  exposures, predictors, potential  confounders, and effect  modifiers. Give diagnostic  criteria, if applicable. | Pages 5-7; Methods, Variables | RECORD 7.1: A complete list of codes and algorithms used to classify exposures, outcomes, confounders, and effect modifiers should be provided. If these cannot be reported, an explanation should be provided. | Pages 5-7; Methods, Variables |
| Data sources/  measurement | 8 | For each variable of interest, give sources of data and details of methods of assessment (measurement). Describe comparability of assessment methods if there is more than one group. | Pages 5-7; Methods, Variables |  | Pages 5-7; Methods, Variables |
| Bias | 9 | Describe any efforts to address potential sources of bias. | Pages 7-9; Methods |  | Pages 7-9; Methods |
| Study size assessment | 10 | Explain how the study size was arrived at | Page 4; Methods. Page 11; data base descriptives. |  |  |
| Quantitative  variables | 11 | Explain how quantitative variables were handled in the analyses. If applicable, describe which groupings were chosen, and why. | Pages 5-7; Methods, Variables |  | Pages 5-7; Methods, Variables |
| Synthesis methods | 12a | Describe all statistical methods, including those used to control for confounding | Pages 7-9; Methods, Statistical Analysis |  |  |
|  | 12b | Describe any methods used to examine subgroups and interactions. | Page 7-9; Methods, Statistical Analysis |  |  |
|  | 12c | Explain how missing data were addressed | Page 5; Methods, Variables |  |  |
|  | 12d | Cohort study - If applicable, explain how loss to follow - up was addressed Case -control study - If applicable, explain how matching of cases and controls was addressed Cross -sectional study - If applicable, describe analytical methods taking account of sampling strategy | Page 7-9; Methods, Propensity score matching, Data Analysis. |  |  |
|  | 12e | Describe any sensitivity analyses | Not applicable |  |  |
| Data access and cleaning methods |  |  |  | RECORD 12.1: Authors should describe the extent to which the investigators had access to the database population used to create the study population.  RECORD 12.2: Authors should provide information on the data cleaning methods used in the study | Page 17. Availability of data and materials  Not applicable |
| Linkage |  |  | - | RECORD 12.3: State whether the study included person -level, institutional -level, or other data linkage across two or more databases. The methods of linkage and methods of linkage quality evaluation should be provided. | Not applicable |
| **RESULTS** | | |  |  |  |
| Participants | 13a | Report the numbers of individuals at each stage of the study (e.g., numbers potentially eligible, examined for eligibility, confirmed eligible, included in the study, completing follow -up, and analysed) | Page 7; Methods, study data. Propensity score matching  Page 10: Results, Cohort Description: Sociodemographics and Most Prevalent Diagnoses.  Supplementary tables 2 and 4. | RECORD 13.1: Describe in detail the selection of the persons included in the study (i.e., study population selection) including filtering based on data quality, data availability and linkage. The selection of included persons can be described in the text and/or by means of the study flow diagram. | Tables in supplementary material. |
|  | 13b | Give reasons for non - participation at each stage. | Page 4; Methods, Study area and period |  |  |
|  | 13c | Consider use of a flow diagram | Not applicable |  |  |
| Descriptive data | 14a | Give characteristics of study participants (e.g., demographic, clinical, social) and information on exposures and potential confounders. | Table 1; Results, population Description: Sociodemographics and Most Prevalent Diagnoses (Table 3) |  |  |
|  | 14b | Indicate the number of participants with missing data for each variable of interest | Table 1; population descriptives, Table S2; markers data availability |  |  |
|  | 14c | Cohort study - summarise follow -up time (e.g., average and total amount) | Not applicable |  |  |
| Outcome data | 15 | Cohort study - Report numbers of outcome events or summary measures over time Case -control study - Report numbers in each exposure category, or summary measures of exposure Cross -sectional study - Report numbers of outcome events or summary measures | Table S4 in supplementary Material. Case- control matching summary. |  |  |
| Main results | 16a | Give unadjusted estimates and, if applicable, confounder - adjusted estimates and their precision (e.g., 95% confidence interval). Make clear which confounders were adjusted for and why they were included. | Page 10; Results. Figures 1 and 2. |  |  |
|  | 16b | Report category boundaries when continuous variables were categorized | Page 10; Results. Figures 1 and 2. |  |  |
|  | 16c | If relevant, consider translating estimates of relative risk into absolute risk for a meaningful time period | - |  |  |
| Other analyses | 17 | Report other analyses done — e.g., analyses of subgroups and interactions, and sensitivity analyses | - |  | - |
| **DISCUSSION** | | |  |  |  |
| Key results | 18 | Summarise key results with reference to study objectives | Pages 12-14, Discussion. |  | Pages 12-14, Discussion. |
| Limitations | 19 | Discuss limitations of the study, taking into account sources of potential bias or imprecision. Discuss both direction and magnitude of any potential bias | Pages 14-15, Discussion. | RECORD 19.1: Discuss the implications of using data that were not created or collected to answer the specific research question(s). Include discussion of misclassification bias, unmeasured confounding, missing data, and changing eligibility over time, as they pertain to the study being reported | Pages 14-15, Discussion. |
| Interpretation | 20 | Give a cautious overall interpretation of results considering objectives, limitations, multiplicity of analyses, results from similar studies, and other relevant evidence | Pages 12-14, Discussion. |  | Pages 12-14, Discussion. |
| Generalisability | 21 | Discuss the generalisability (external validity) of the study results | Page 16, Discussion |  | Page 16, Discussion |
| **OTHER INFORMATION** | | |  |  |  |
| Funding | 22 | Give the source of funding and the role of the funders for the present study and, if applicable, for the original study on which the present article is based | Page 9; Role of the Funding Source  Pages 17-18; Funding |  |  |
| Accessibility of protocol, raw data, and programming code |  | Declare any competing interests of review authors. | Page 17; Competing interests | RECORD 22.1: Authors should provide information on how to access any supplemental information such as the study protocol, raw data, or programming code. | Page 17; Availability of data and materials |

1.- Benchimol EI, Smeeth L, Guttmann A, et al. The REporting of studies Conducted using Observational Routinely-collected health Data (RECORD) statement. *PLoS Med.* 2015; 12. DOI:10.1371/JOURNAL.PMED.1001885.

**Table S2.- Specifications of the priors of the random effects.**

Priors for the Matérn covariance function of the spatially structured random effect:

$$Cov\left( S\left( x_{i} \right),S\left( x_{i^{'}} \right) \right)=\frac{\sigma^{2}}{2^{\nu-1}\Gamma\left( \vartheta\right)} \left( \kappa\left\| x_{i}-x_{i^{'}} \right\| \right)^{\vartheta} K_{\vartheta} \left( \kappa\left\| x_{i}-x_{i^{'}} \right\| \right)$$

where $K_{\vartheta}$ is the modified Bessel function of the second type and order $\vartheta>0$. $\vartheta$ is a smoother parameter; $\sigma^{2}$ is the variance; and $\kappa>0$ is related to the range ($\rho=\sqrt{8 \vartheta}/\kappa$), the distance to which the spatial correlation is close to 0.1 (Lindgren *et al.,* 2011).

Prior for $\sigma$ (i.e. Standard deviation) $Prob\left( \sigma>100 \right)=0.1$

Prior for $\rho$ (i.e. range) $Prob\left( \rho<0.01 \right)=0.01$,

Prior for the precision (i.e. inverse of the variance) of the (unstructured) random effects indexed on the matched case-control pair

$Prob\left( \sigma>0.5 \right)=0.01$,

where $\sigma=1/\sqrt{precision}$

Lindgren FK, Rue H, Lindström J. An explicit link between Gaussian fields and Gaussian Markov random fields: the stochastic partial differential equation approach. *J R Stat Soc Series B Stat Methodol*. 2011; 73(4):423-498. doi: [j.1467-9868.2011.00777.x](file:///Users/marcsaez/Documentos/Treballs%20varis/COVID-19/Resultados/Vaccination/j.1467-9868.2011.00777.x).

**Table S3.- Biomarker records availability by subperiods.**

|  | **Proportion of individuals** | | | **Number of individuals** | |
| --- | --- | --- | --- | --- | --- |
|  | **No marker** | **Marker in a subperiod** | **Markers in both subperiods** | **Markers Pre-pandemic** | **Markers Pandemic** |
| **Anaemia** |  |  |  |  |  |
| Iron | 14,531(56·2%) | 10,147 (39·2%) | 1,194 (4·6%) | 10,741 | 1,794 |
| Haemoglobin | 3,368 (13·0%) | 18,777 (72·6%) | 3,727 (14·4%) | 22,368 | 3,863 |
| Ferritin | 10,620 (41·0%) | 13,184 (51·0%) | 2,068 (8·0%) | 14,654 | 2,666 |
| **Lipids** |  |  |  |  |  |
| LDL-C | 10,579 (40·9%) | 13,429 (51·9%) | 1,864 (7·2%) | 15,027 | 2,130 |
| HDL-C | 10,574 (40·9%) | 13,433 (51·9%) | 1,865 (7·2%) | 15,032 | 2,131 |
| Total Cholesterol | 5,022 (19·4%) | 17,699 (68·4%) | 3,151 (12·2%) | 20,674 | 3,327 |
| **Glycemia** |  |  |  |  |  |
| HbA1c | 18,127 (70·1%) | 6,961 (26·9%) | 784 (3·0%) | 7,479 | 1,050 |
| Fasting blood glucose | 3,736 (14·4%) | 18,527 (71·6%) | 3,609 (13·9%) | 22,004 | 3,741 |
| **Kidney function** |  |  |  |  |  |
| CKD-EPI | 3,821 (14·8%) | 18,413 (71·2%) | 3,638 (14·1%) | 21,879 | 3,810 |
| Creatinine | 3,604 (13·9%) | 18,678 (72·2%) | 3,590 (13·9%) | 22,134 | 3,724 |
| Urea | 13,091 (50·6%) | 11,412 (44·1%) | 1,369 (5·3%) | 12,254 | 1,896 |
| **Liver function** |  |  |  |  |  |
| ALP | 16,006 (61·9%) | 8,885 (34·3%) | 981 (3·8%) | 9,338 | 1,509 |

HDL-C: high-density lipoprotein cholesterol; LDL-C low-density lipoprotein cholesterol; HbA1c: glycosylated haemoglobin; CKD-EPI: glomerular filtration rate; ALP: alkaline phosphatase

Total number of individuals: 25,872

**Table S4.- Improvement in the standardized variation of the biomarker before and during the pandemic.**

| **Improvement in the biomarker** | **Non-centenarians^[1]^** | **Centenarians^[1]^** |
| --- | --- | --- |
| Anaemia biomarkers |  |  |
| Iron (n=885) | 22 (17·9%) | 162 (21·3%) |
| Haemoglobin (n=2,757) | 84 (22·5%) | 498 (20·9%) |
| Ferritin (n=1,516) | 24 (11·2%) | 122 (9·4%) |
| Cholesterol biomarkers |  |  |
| Total cholesterol (n=2,372) | 59 (19·9%) | 451 (21·7%) |
| HDL-C (n=1,414) | 37 (21·3%) | 275 (22·2%) |
| LDL-C (n=1, 414) | 38 (21·8%) | 268 (21·6%) |
| Glycemia biomarkers |  |  |
| HbA1c (n=602) | 16 (19·0%) | 74 (14·3%) |
| Fasting blood glucose (n=2,690) | 44 (12·5%) | 274 (11·7%) |
| Kidney function biomarkers |  |  |
| CKD-EPI (n=2,687) | 72 (19·9%) | 497 (21·4%) |
| Urea (n=979) | 23 (17·0%) | 127 (15·0%) |
| Creatinine (n=2,664) | 43 (12·3%) | 301 (13·0%) |
| Liver functioning biomarker |  |  |
| ALP (n=722) | 7 (7·4%) | 48 (7·6%) |

HDL-C: high-density lipoprotein cholesterol; LDL-C low-density lipoprotein cholesterol; HbA1c: glycosylated haemoglobin; CKD-EPI: glomerular filtration rate; ALP: alkaline phosphatase

[1] n (%)

**Table S5.- Matching summary for centenarians in the pre-pandemic period.**

|  | **Non-Centenarian** | **Centenarian** |
| --- | --- | --- |
|  |  |  |
| Individuals with markers measured only in pre-pandemic period | 17,307 (81·6%) | 3,890 (18·3%) |
| **Matched** | 3,890 (22·4%) | 3,890 (100%) |
| **Unmatched** | 13,417 (77·6%) | 0 |

**Matching summary for centenarians with markers during the pandemic period.**

|  | **Non-Centenarian** | **Centenarian** |
| --- | --- | --- |
|  |  |  |
| Individuals with markers in pre-pandemic and pandemic periods | 18,444 (83·9%) | 3,538 (16%) |
| **Matched** | 2,977 (16·1%) | 2.977 (84·1%) |
| **Unmatched** | 15,467 (83·8%) | 561 (15·8%) |

**Table S6.- Descriptive data on biomarkers in centenarian individuals. Latest blood test available^[1]^.**

| **Biomarker** | **Living centenarians** | **Centenarians dead** | |
| --- | --- | --- | --- |
|  |  | **Before April 2020** | **After March 2020** |
| Anaemia biomarkers |  |  |  |
| Iron | 56.58 (25.48)  59 [44.70, 74.93] | 55.17 (27.47)  53.00 [36.00, 71.00] | 63.87 (27.40)  62.00 [44.00, 82.40] |
| Haemoglobin | 11.50 (1.80)  11.60 [10.30,12.60] | 11.39 (1.97)  11.50 [10.20, 12.70] | 11.60 (1.78)  11.40 [10.35,13.00] |
| Ferritin | 180.14 (164.75)  133.30 [56,30, 276.00] | 180.94 (236.93)  111.00 [49.16, 227.45] | 197.81 (222.25)  123.40 [55.55, 257.25] |
| Cholesterol biomarkers |  |  |  |
| Total cholesterol | 169.23 (42.61)  164.00 [140.75,196.50] | 165.19 (44.40)  162.00 [135.00, 192.00] | 149.74 (30.32)  144.0 [133.50,165.00] |
| HDL-C | 47.49 (12.41)  46.50 [38.00, 53.25] | 46.57 (13.29)  45.00 [37.00, 54.00] | 43.79 (9.41)  43.00 [37.00, 50.00] |
| LDL-C | 100.71 (35.61)  96.50 [80.00, 117.50] | 97.12 (33.10)  93.00 [74.00, 118.00] | 85.96 (25.93)  83.00 [67.00, 102,50] |
| Glycemia biomarkers |  |  |  |
| HbA1c | 6.16 (1.22)  5.85 [5.50, 6.50] | 6.30 (1.21)  5.90 [5.50, 6.80] | 6.06 (0.96)  5.80 [5.40, 6.35] |
| Fasting blood glucose | 104.10 (41.03)  89.00 [80.00, 115.25] | 117.56 (56.28)  100.00 [83.00, 132.00 | 110.19 (51.93)  88.00 [77.50, 132.00] |
| Kidney function biomarkers |  |  |  |
| CKD-EPI | 42.27 (18.49)  46.75 [32.55, 61.70] | 44.59 (20.08)  42.10 [28.85, 57.90] | 47.08 (21.48)  43.80 [30.50, 68.95] |
| Urea | 63.92 (40.98)  58.50 [32.,65, 73.00] | 69.12 (47.25)  61.00 [42.15, 88.50] | 66.00 (41.49)  58.00 [37.40, 76.55] |
| Creatinine | 1.17 (0.50)  1.05 [0.80, 1.45] | 1.38 (0.77)  1.18 [0.90, 1.62] | 1.27 (0.75)  1.18 [0.71, 1.44] |
| Liver functioning biomarker |  |  |  |
| ALP | 99.49 (55.78)  86.50 [66.00, 114.00] | 105.43 (67.21)  89.00 [71.00, 116.00] | 109.49 (77.10)  86.00 [71.00, 122.50] |

HDL-C: high-density lipoprotein cholesterol; LDL- low-density lipoprotein cholesterol; HbA1c: glycosylated haemoglobin; CKD-EPI: glomerular filtration rate; ALP: alkaline phosphatase

[1] First row: Mean (standard deviation)

Second row: Median [Q1,Q3]

**Table S7.- Improvement in the standardized variation of the biomarker before and during the pandemic for cut-off points at the 10/90th and 5/95th percentiles.**

**Cut-off at the 10^th^/90 ^th^ percentiles**

| **Improvement in the biomarker** | **Non-centenarians^[1]^** | **Centenarians^[1]^** |
| --- | --- | --- |
| **Anaemia biomarkers** |  |  |
| Iron (n=885) | 10 (8.1%) | 66 (8.7%) |
| Haemoglobin (n=2,757) | 31 (8.3%) | 178 (7.5%) |
| Ferritin (n=1,516) | 11 (5.1%) | 65 (5.0%) |
| **Cholesterol biomarkers** |  |  |
| Total cholesterol (n=2,372) | 26 (8.8%) | 189 (9.1%) |
| HDL-C (n=1,414) | 10 (5.7%) | 86 (6.9%) |
| LDL-C (n=1, 414) | 10 (5.7%) | 90 (7.3%) |
| **Glycemia biomarkers** |  |  |
| HbA1c (n=602) | 8 (9.5%) | 33 (6.4%) |
| Fasting blood glucose (n=2,690) | 17 (4.8%) | 103 (4.4%) |
| **Kidney function biomarkers** |  |  |
| CKD-EPI (n=2,687) | 26 (7.2%) | 168 (7.2%) |
| Urea (n=979) | 6 (4.4%) | 28 (3.3%) |
| Creatinine (n=2,664) | 11 (3.1%) | 54 (2.3%) |
| **Liver functioning biomarker** |  |  |
| ALP (n=722) | 4 (4.3%) | 14 (2.2%) |

**Cut-off at the 5^th^/95 ^th^ percentiles**

| **Improvement in the biomarker** | **Non-centenarians^[1]^** | **Centenarians^[1]^** |
| --- | --- | --- |
| **Anaemia biomarkers** |  |  |
| Iron (n=885) | 5 (4.1%) | 33 (4.3%) |
| Haemoglobin (n=2,757) | 21 (5.6%) | 95 (4.0%) |
| Ferritin (n=1,516) | 11 (5.1%) | 44 (3.4%) |
| **Cholesterol biomarkers** |  |  |
| Total cholesterol (n=2,372) | 16 (5.4%) | 111 (5.3%) |
| HDL-C (n=1,414) | 3 (1.7%) | 36 (2.9%) |
| LDL-C (n=1, 414) | 8 (4.6%) | 52 (4.2%) |
| **Glycemia biomarkers** |  |  |
| HbA1c (n=602) | 5 (6.0%) | 19 (3.7%) |
| Fasting blood glucose (n=2,690) | 11 (3.1%) | 65 (2.8%) |
| **Kidney function biomarkers** |  |  |
| CKD-EPI (n=2,687) | 18 (5.0%) | 78 (3.4%) |
| Urea (n=979) | 2 (1.5%) | 10 (1.2%) |
| Creatinine (n=2,664) | 5 (1.4%) | 17 (0.7%) |
| **Liver functioning biomarker** |  |  |
| ALP (n=722) | 3 (3.2%) | 7 (1.1%) |

**Figure S1. Matching balancing result for pre-pandemic centenarians.**

**
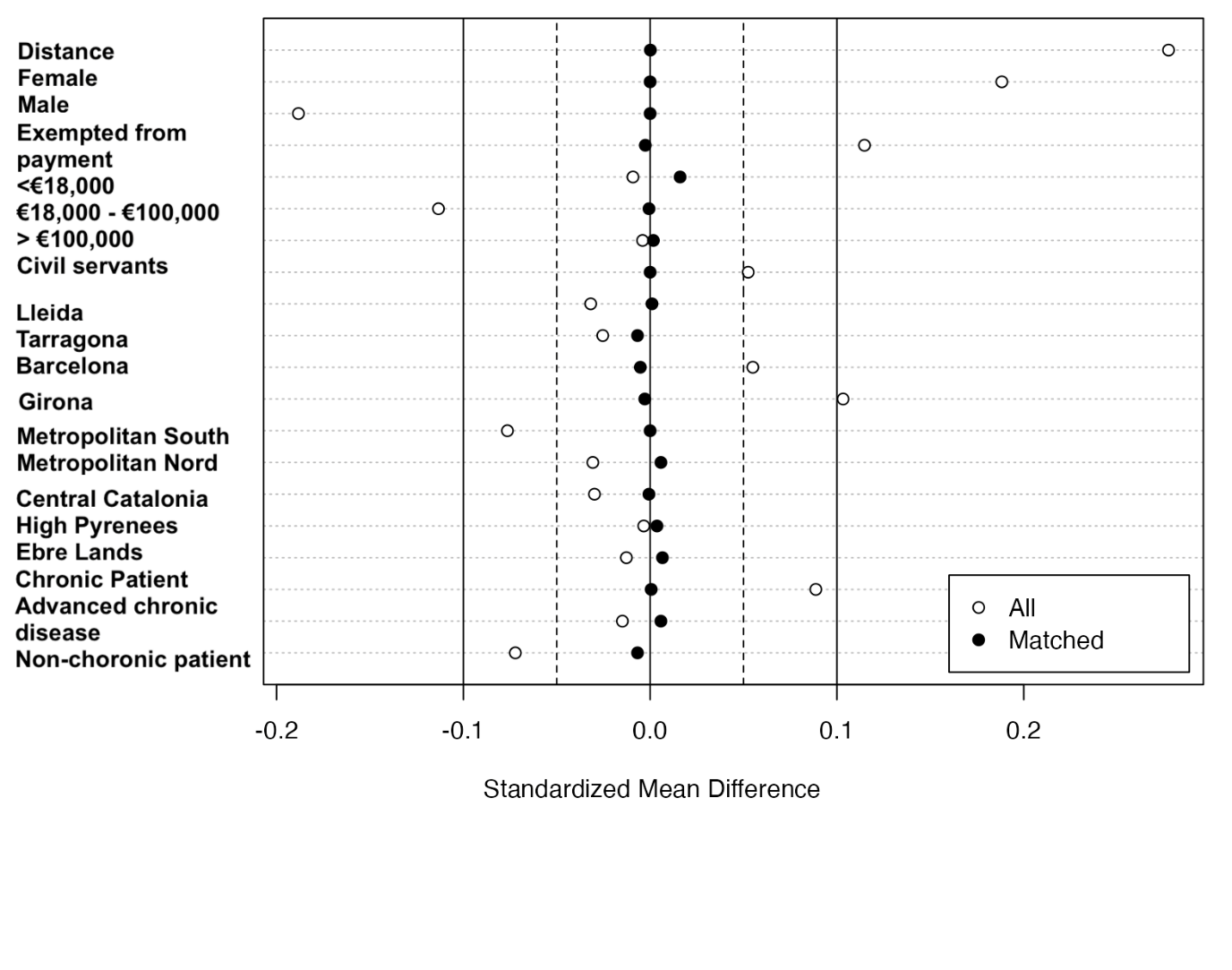
**

**Figure S2. Matching balancing results in centenarians during the pandemic period.**

**
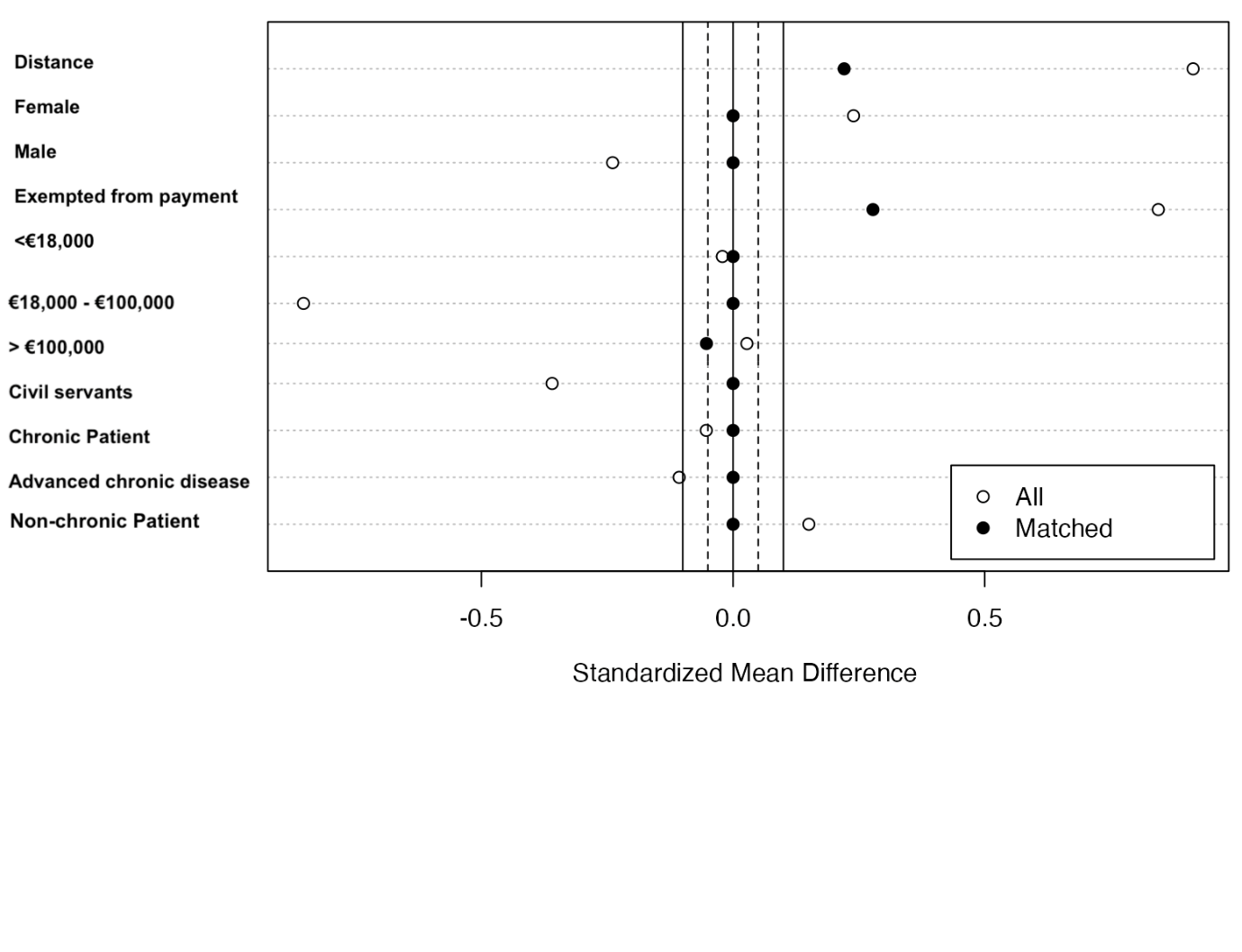
**

**Figure S3.- Results of assessing how variations in the biomarkers during the COVID-19 pandemic influenced the probability of attaining centenarian age.**

**Cut-off at the 10^th^/90 ^th^ percentiles**


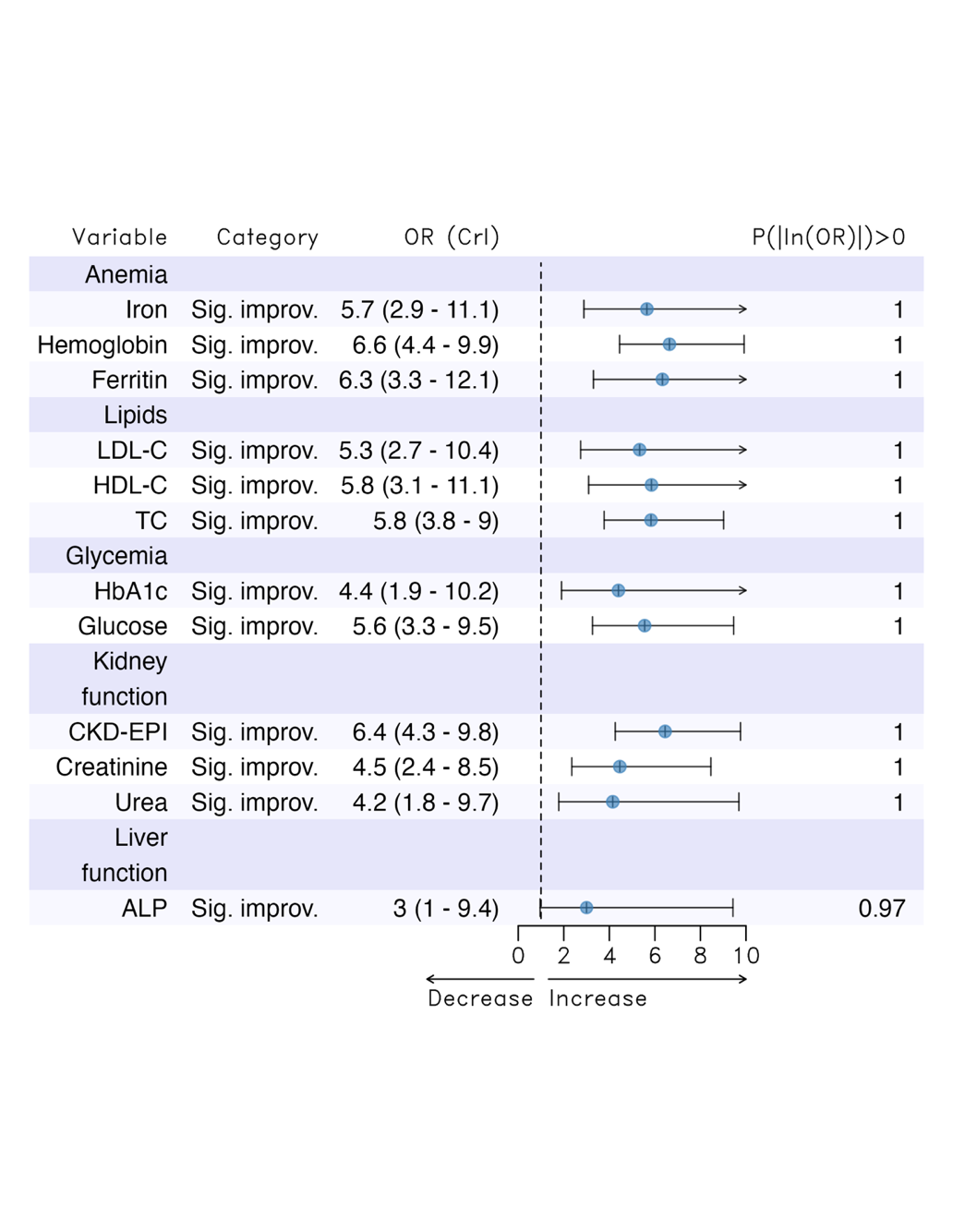


**Cut-off at the 5^th^/95 ^th^ percentiles**


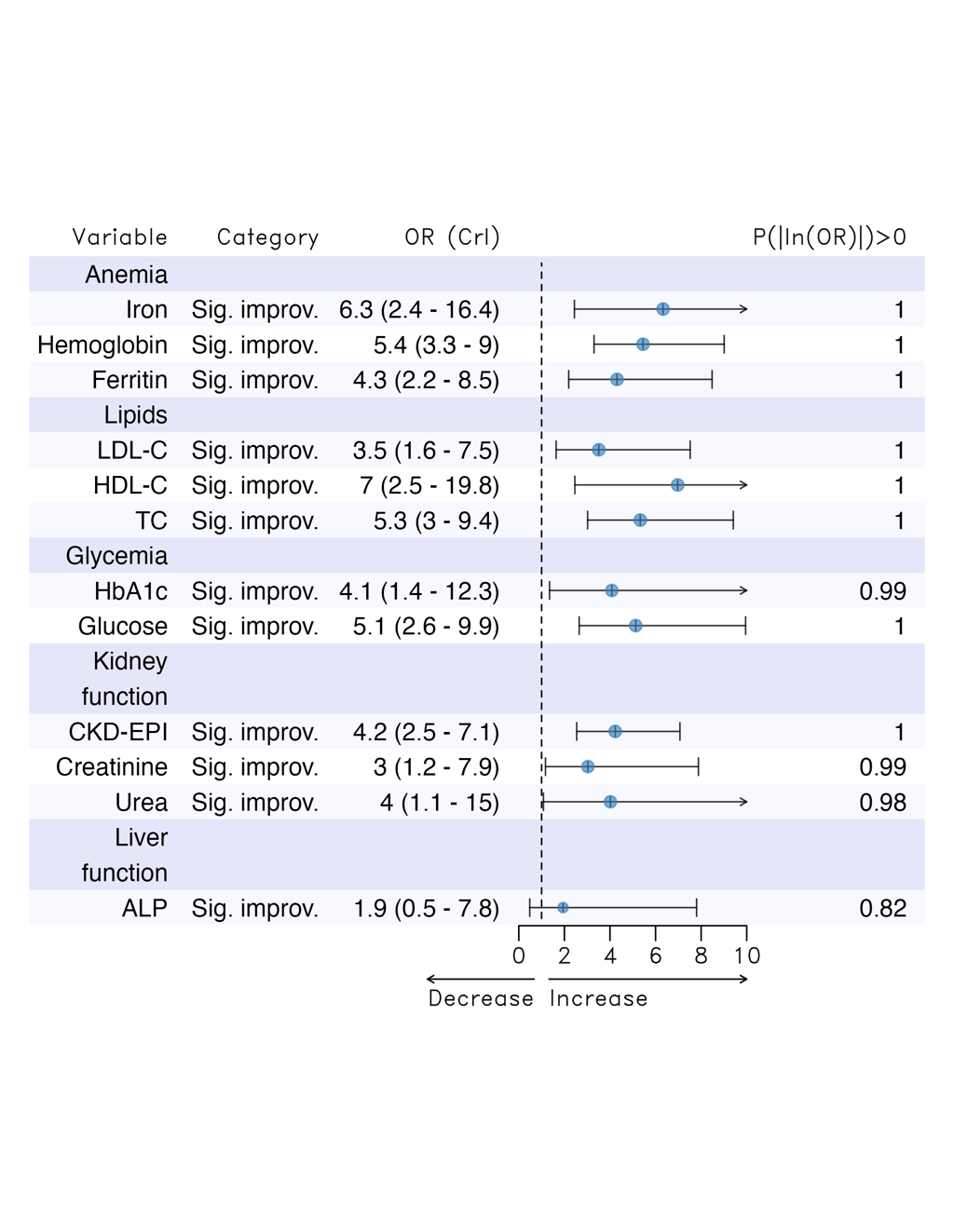

Supplement: Supplementary file 1 — Supplementary file1 (DOCX 768 KB) [file 10522_2025_10258_MOESM1_ESM.docx]
